# Circular RNA as an Epigenetic Regulator in Chronic Liver Diseases

**DOI:** 10.3390/cells10081945

**Published:** 2021-07-30

**Authors:** Xianhui Zeng, Xianglei Yuan, Qiuyu Cai, Chengwei Tang, Jinhang Gao

**Affiliations:** 1 Lab of Gastroenterology and Hepatology, West China Hospital, Sichuan University, No. 1, 4th Keyuan Road, Chengdu 610093, China; ruading@163.com (X.Z.); caiqiuyu97@163.com (Q.C.); shcqcdmed@163.com (C.T.); 2 Department of Gastroenterology, West China Hospital, Sichuan University, Chengdu 610041, China; yuanxianglei94@163.com

**Keywords:** circular RNA, epigenetics, alcoholic liver disease, metabolic-associated fatty liver disease, nonalcoholic fatty liver disease, nonalcoholic steatohepatitis, hepatitis, liver regeneration, liver cirrhosis, autoimmune liver disease

## Abstract

Circular RNA (circRNA) is a type of non-coding RNA characterized by a covalently closed continuous loop. CircRNA is generated by pre-mRNA through back-splicing and is probably cleared up by extracellular vesicles. CircRNAs play a pivotal role in the epigenetic regulation of gene expression at transcriptional and post-transcriptional levels. Recently, circRNAs have been demonstrated to be involved in the regulation of liver homeostasis and diseases. However, the epigenetic role and underlying mechanisms of circRNAs in chronic liver diseases remain unclear. This review discussed the role of circRNAs in non-neoplastic chronic liver diseases, including alcoholic liver disease (ALD), metabolic-associated fatty liver disease (MAFLD), viral hepatitis, liver injury and regeneration, liver cirrhosis, and autoimmune liver disease. The review also highlighted that further efforts are urgently needed to develop circRNAs as novel diagnostics and therapeutics for chronic liver diseases.

## 1. Introduction

The liver is a critical organ for humans. It is composed of hepatocytes, biliary epithelial cells (cholangiocytes), hepatic stellate cells (HSCs), Kupffer cells, mast cells, and liver sinusoidal endothelial cells (LSECs). These cells cooperatively regulate hepatic function in numerous physiological processes, including glucose metabolism, lipid and cholesterol homeostasis, maintenance of energy balance, blood volume regulation, immune responses, and detoxification [1,2]; intercellular communication also contributes to the pathogenesis of liver diseases [3,4,5]. The liver has a powerful and unique regenerative capacity to maintain the constant size required for body homeostasis, regardless of injury [6,7]. However, various pathogenic agents can damage the liver, resulting in acute or chronic liver disease, such as alcoholic liver disease (ALD), metabolic-associated fatty liver disease (MAFLD), viral hepatitis, hepatocellular carcinoma (HCC), and their complications [8,9]. Liver cirrhosis accounts for approximately one million deaths worldwide annually [10]. The leading causes of cirrhosis in Western countries are alcohol and MAFLD, whereas viral hepatitis B is the main cause in China and other Asian countries [8]. However, the pathogenesis of liver diseases is still not fully understood.

Epigenetic research has accelerated rapidly in the past two decades, and achievements in epigenetics have advanced our knowledge of the pathogenesis of liver diseases [11,12,13]. In addition, epigenetic modifications have a significant impact on regulating gene expression and occurrence, and the development of diseases [14]. The major carriers of epigenetic information include non-coding RNAs, DNA methylation, histone acetylation, heterochromatin components, and polycomb proteins [15].

Among non-coding RNAs, microRNA (miRNA), long non-coding RNA (lncRNA) and circular RNA (circRNA) are the most well-studied non-coding RNAs. miRNAs are non-coding RNAs of only 20–22 nucleotides in length; they repress gene expression by the RNA-induced silencing complex (RISC) [16]. A single miRNA can correspond to many different mRNAs, whereas a single mRNA is regulated by multiple miRNAs [17]. lncRNAs are considered to be longer than 200 nucleotides, which is distinctly different from mRNAs, miRNAs, small nuclear RNAs (snoRNAs) and tRNAs. They are able to regulate gene expression at the level of chromatin modification, transcription and post-transcriptional processing through a diversity of mechanisms [16,18]. The ability of lncRNAs to regulate associated protein-coding genes might contribute to many diseases. The competitive endogenous RNA (ceRNA) hypothesis is a theory about lncRNA function that is gaining significant attention; it states that competition for miRNA binding sites regulates the expression of a target gene. The role of lncRNA in chronic liver diseases has previously been described and is not the focus of this review [19,20,21,22].

**Figure 1:**
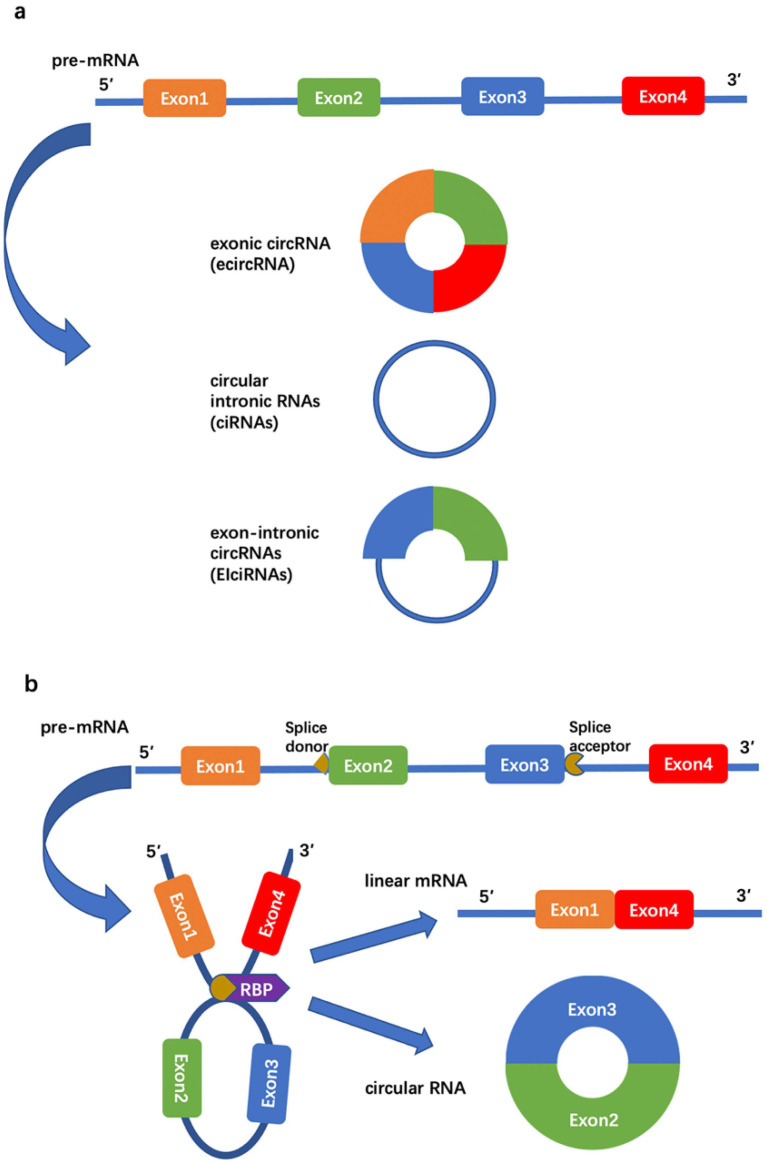
Formation of circRNAs. (**a**) Three types of circRNAs according to their sources of pre-mRNA. (**b**) RNA-binding protein (RBP) induces circulation. Circular exons are generated by pre-mRNA through back-splicing between the splice donor site of a downstream exon (the 5′ end of exon 2) and the splice acceptor site of an upstream exon (the 3′ end of exon 3). This event can be mediated by RBPs or specific sequence elements, resulting in the production of a circular RNA and a linear RNA. Blue lines represent introns.

circRNAs are characterized by a covalently closed continuous loop [23]. They have the advantages of stability, conservation, and tissue specificity [24], generated by pre-mRNA through back-splicing (Figure 1).

Degradation of circRNAs cannot be achieved through many classical RNA degradation pathways. They might be eliminated from cells by exocytosis [23,24,25]. Enclosing circRNAs or circRNA complexes in vesicles and releasing them into the extracellular space is a way to move circRNAs out of the cytoplasm. However, with the protection of vesicles, circRNAs or circRNA complexes can reach other cells or tissues to act as messenger molecules or to fulfill other unknown functions [23]. The degradation of circRNA theoretically involves endonuclease. A recent study found that the N6-methylation of adenosine (m^6^A) modification facilitates the recruitment of endonucleases, affecting the degradation of circRNAs via a YTHDF2-HRSP12-RNase P/MRP axis [26].

**Figure 2:**
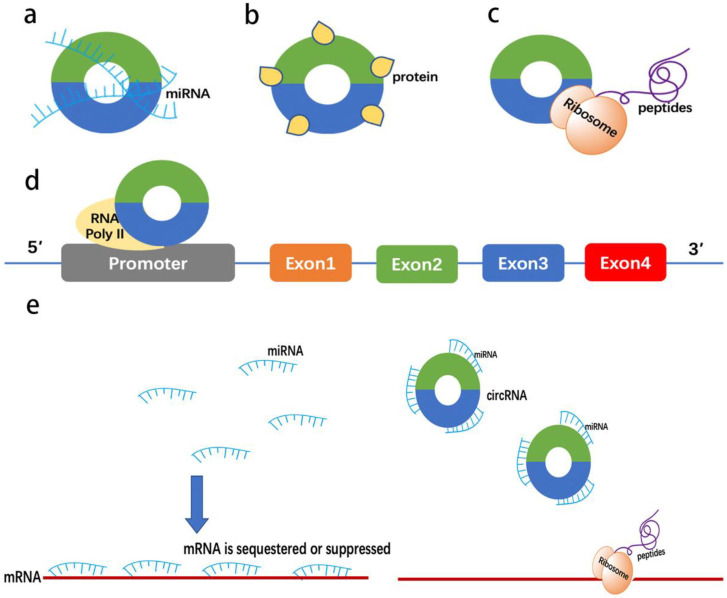
The main functions of circRNAs. (**a**) miRNA sponge; (**b**) protein sponge; (**c**) translating into peptides; (**d**) regulating the expression of the parental gene via interacting with RNA polymerase II and regulating transcription; (**e**) miRNA sponges alleviate the inhibitory effects of miRNAs on target mRNA.

circRNAs act through several mechanisms (Figure 2), including: (1) miRNA sponges or ceRNA mechanisms [16], which alleviate the inhibitory effects of miRNAs on target genes, thereby increasing the levels of the target genes [27]; (2) protein sponge, such as sponging RNA-binding protein components (RBPs); (3) regulating the expression of a parental gene; and (4) translating into peptides [24,28,29].

Through novel bioinformatics approaches integrated with biochemical enrichment strategies and deep sequencing, circRNAs can be studied in a comprehensive and in-depth perspective [30,31]. Many online resources have been developed, such as identification tools, databases, detection software systems, websites, and recommended nomenclature of circRNAs [28,32,33,34]. Gene Ontology (GO) and the Kyoto Encyclopedia of Genes and Genomes (KEGG) pathway have generally been used to reveal the potential role of the differentially expressed circRNAs. As a rising star in the past few years, circRNAs have been proven to be involved in regulating liver homeostasis and diseases [35]. A certain number of circRNAs have been discovered as potential biomarkers or therapeutic targets for HCC [36,37,38,39,40,41,42,43]. As well as for HCC, circRNAs play an important role in non-neoplastic chronic liver diseases, such as ALD, MAFLD, liver fibrosis, and autoimmune diseases through epigenetic mechanisms [11,12,44,45,46].

Here, we conduct a literature review to summarize the role of circRNAs in ALD, MAFLD, viral hepatitis, liver injury and regeneration, liver cirrhosis, and autoimmune liver disease.

## 2. Circular RNAs in Chronic Liver Diseases

### 2.1. Alcoholic Liver Disease (ALD)

ALD, ranging from asymptomatic liver steatosis to alcoholic hepatitis and cirrhosis, is the most prevalent type of chronic liver disease worldwide [47]. ALD is characterized by fat accumulation, hepatocyte apoptosis, and inflammatory infiltration [48]. The pathogenesis of ALD involves several factors, including lipid deposition, excessive generation of reactive oxygen species (ROS), hepatocyte apoptosis, increased gut permeability, translocation of bacteria-derived lipopolysaccharides (LPS) from the gut into the liver, and excessive inflammatory responses that share some relationships with miRNAs and circRNAs [47,49,50].

miR-122 is one of the most well-studied miRNAs. The target genes of miR-122 include genes that are related to lipid metabolism (*HMGCG*, *ApoE*, *MTTP*, and *PGC1a*), liver fibrosis (*P4HA1*), hepatocellular cancer-relevant genes (*Igf1R*, *ADAM10*, *cyclin G1*, and *KLF6*) [51], hypoxia-related genes (*HIF1α*) [52], and inflammatory responses-associated genes (*TNFRSF13C*) [49]. miR-122 is a suppressive factor in ALD, and is sponged by circRNA_1639. circRNA_1639 is a pro-inflammatory factor identified in primary Kupffer cells in CCl_4_-induced liver fibrosis. circRNA_1639 induces ALD via activating the NF-κB signaling pathway through circRNA_1639/miR-122/TNFRSF13C axis [49]. In other studies of alcohol-induced animal models, mm9_circ_018725 [53] and mou_circ_1657 [54] have contributed to ALD by releasing pro-inflammatory cytokines and activating HSCs via interacting with miR-96-5p, respectively. miR-21 also plays a protective role against ALD through the anti-extrinsic apoptotic signaling pathway [55], but no circRNA corresponding to it under the ceRNA mechanism has been reported yet.

Another pivotal miRNA, miR-155, is a crucial regulator for inflammation and is increased in the liver and circulation in ALD [47]. Kupffer cell activation and sensitization to LPS are regulated by miRNA-155 in ALD [51]. Bioinformatics analysis predicted that circRNA-0067835 acts as an miR-155 sponge to regulate FOXO3a expression in the progress of liver fibrosis [56]. However, there is no report in which circRNAs specifically interact with miR-155 in ALD.

In summary, ALD is caused by various factors, including genetics, epigenetics, and non-genetic factors. The ceRNA mechanism might also play an essential role in the development of ALD, an area which needs further study. Additionally, alcohol intake can lead to changes in the composition of the gut microbiota. Functional alterations in the microbiota have been proven to be associated with ALD progression [50]. Similarly, gut microbiota regulates tumor lung metastasis via a circRNA/miRNA network [57]. Accordingly, gut microbiota might also interact with the circRNA/miRNA network in ALD, making it a promising research direction.

### 2.2. Metabolic-Associated Fatty Liver Disease (MAFLD)

Nonalcoholic fatty liver disease (NAFLD) has become the most common cause of chronic liver diseases [58]. Nonalcoholic steatohepatitis (NASH), currently recognized as part of NAFLD, progresses into liver cirrhosis and further decompensation liver cirrhosis in some individuals [59]. Recently, international experts reached a consensus that the disease abbreviation should be changed from NAFLD to metabolic dysfunction-associated fatty liver disease (MAFLD) [60]. Insulin resistance and abnormal lipid metabolism [61] are the hallmarks of MAFLD, and many circRNAs have effects on oxidative stress, including mitochondrial dysfunction and endoplasmic reticulum (ER) stress [62], and on autophagy or lipophagy in fatty livers [63]. For example, *LPIN1* is a crucial gene in regulating triglyceride and phospholipid biosynthesis [64]. The circRNA_021412/miR-1972/LPIN1 signaling pathway reflected the regulatory mechanisms underlying the steatosis-related circRNA–miRNA–mRNA network [65]. CircScd1 promotes fatty liver disease by increasing hepatocellular lipidosis in NAFLD via the JAK2/STAT5 pathway [66]. Low-density lipoprotein (LDL), peroxisome proliferator-activated receptor α (PPARα), and their corresponding genes are critical in lipometabolisim. circRNA_0049392 is differentially expressed in an NAFLD mouse model [67], whereas target miRNAs of circRNA_0049392 are miR-6919-5p and miR-7037-5p, which are validated to regulate LDL [67]. miR-34a, an inhibitor of PPARα, is significantly up-regulated in different models of rodent hepatic steatosis. circRNA_0046366 and circRNA_0046367, as the sponges of miR-34a, abolish the inhibitory effect of miR-34a on PPARα, thus ameliorating the lipoxidative stress [68,69]. The circRNA_002581/miR-122/CPEB1 axis promoting NASH is associated with the dysregulation of autophagy, which can be inhibited through the PTEN/AMPK/mTOR signaling pathway [70].

Liver fibrosis is also recognized as the hallmark of disease progression in NASH [58], and fibroblasts, along with HSCs, contribute to the occurrence and development of liver fibrosis. circRNA_29981, a potential regulator of HSC activation, was identified in the murine NASH model [71]. Moreover, a steatohepatitis-associated mitochondrial circRNA-SCAR can shut down the mitochondrial permeability transition pore (mPTP) by binding to ATP5B, thereby inhibiting mitochondrial ROS (mtROS) output and fibroblast activation [62]. It seems that circRNAs might also be involved in the progress of NASH to liver fibrosis. circRNAs are also involved in the efficacy and mechanism of NAFLD treatment. Metformin is now recommended for NAFLD treatment to improve insulin resistance in NAFLD [58]. Metformin systematically alleviates the transcriptome alterations induced by a high-fat diet, while the circRNA–miRNA–mRNA network may attribute to the beneficial effect of metformin on NAFLD [72]. The traditional Chinese medicine prescription, Qianggan formula, has also been reported to improve NASH by modulating the lncRNA/circRNA immune ceRNA network [73].

Altogether, there is increasing evidence suggesting that circRNAs, as well as ceRNA mechanisms, play a crucial role in the development and progression of MAFLD. It is a network where advances in the study of one molecule may provide ideas for the study of other disease mechanisms. For example, miR-34a also functions as a profibrotic factor that promotes alcohol-induced liver fibrosis by reducing HSC senescence and increasing the senescence of hepatocytes [74]. It is possible that in ALD, miR-34a could interact with certain circRNAs to promote or inhibit disease progression. Further studies are needed to deepen the mechanism.

### 2.3. Viral Hepatitis (Hepatitis B and Hepatitis C)

Chronic hepatitis B virus (HBV) infection is one of the leading causes of liver fibrosis, cirrhosis, and hepatocellular carcinoma [75,76]. Interferons (IFNs) are used in anti-HBV therapy. Recently, Zhang et al. found that the circRNA hsa_circ_0004812 could regulate the levels of IFNs and thus their anti-HBV efficiency [77]. Knockdown of hsa_circ_0004812 in HBV-infected cells resulted in a significant increase in mRNA and protein levels of IFN-α and IFN-β [77]. TGFβ2 has suppressive effects on IL-2-dependent T-cell growth and is significantly down-regulated in chronic hepatitis B patients [78]. Zhou et al. found a strong positive correlation between hsa_circ_0000650 and TGFβ2 in the liver biopsies from chronic hepatitis B patients compared with the control group, whereas a robust negative correlation between hsa_circ_0000650 and miR-6873-3p was also established [79].

Hepatitis C virus (HCV) is also an important pathogen of hepatitis, cirrhosis and HCC. HCV is hepatocyte-specific, partly due to its dependence on the host miR-122, which is exceptionally abundant in hepatocytes [80]. miR-122 is thought to enhance the viral RNA abundance and has already been utilized as a drug target for anti-HCV therapy [81]. Jost et al. designed artificial circRNAs containing an array of miR-122 binding sites, thus efficiently sequestering miR-122 [82]. They also found that these circRNAs might work as protein sponges containing binding sites derived from SELEX- or CLIP-data, and were available for multiple RNA-binding proteins [82]. Besides, circPSD3, inhibiting viral RNA abundance at a post-translational step, was shown to promote HCV infection [83].

As circRNAs are more stable than linear RNAs, they might play crucial roles in viral infection pathogenesis. Interestingly, there are also many covalently closed circular DNAs (cccDNA) in the genome of the hepatitis virus itself. For example, HBV cccDNA produced during HBV replication is predominant in the liver of CHB patients [84], and their levels can be regulated by the RNA-binding protein, DHX9 [85]. However, whether the circRNA originated from cccDNA remains unclear.

### 2.4. Liver Injury and Liver Regeneration

The liver has a remarkable capacity for regeneration when stimulated with physical (loss of volume or ischemia) or chemical (medicine or infection) damage [86]. In the 2/3 partial hepatectomy (PH) murine model, a ceRNA network containing 5 circRNAs, 28 target genes, and 533 miRNAs was identified during the DNA synthesis phase (36 h) of liver regeneration [87]. Knockdown of circ_0002498, circ_0000281, and circ_0003822 significantly restrained the proliferation of hepatocytes. In contrast, the knockdown of circ_0003745 and circ_0004245 accelerated the proliferation of hepatocytes [87]. Similarly, a circRNA–miRNA interactions network was also established in the rat PH model. Four significantly changed circRNAs (circ432, circ2077, circ1366, and circ15) were screened as key circRNAs during the priming phase of liver regeneration. These circRNAs might regulate the rat liver regeneration by controlling the expression of *MAPK14*, *FN1*, *TNFRSF21*, and *GOT1*, respectively [88].

Hepatic ischemia and reperfusion injury (IRI) are major complications during surgical procedures such as liver transplantation, liver resection, and trauma surgery [89]. The comprehensive analysis of circRNAs during hepatic IRI was performed using next-generation RNA sequencing [90,91], and several circRNAs were selected as potential targets against hepatic IRI. In the hepatic IRI model established by hepatic artery occlusion, circRNA_017753–miR-218-5p–Jade1, circRNA_017753–miR-7002-3p–Jade1, and circRNA_017753–miR-7008-3p–Jade1 ceRNA signaling pathways may play important roles in the mechanisms of ischemic preconditioning protection [92].

For the radiation-induced liver injury, circRSF1 was upregulated in irradiated LX2 cells, and promoted inflammatory and fibrotic phenotypes of HSC by sponging miR-146a-5p [93]. Similarly, circTUBD1 acted as an miR-146a-5p sponge to affect the viability and pro-inflammatory cytokine production of LX-2 cells through the Toll-like receptor 4 (TLR4)/NF-κB signaling pathway [94]. These two studies suggest that circRSF1 and circTUBD1 are potential targets for radiation-induced liver disease therapy.

Drug-induced liver injury is one of the major causes of liver disease. For acetaminophen (APAP)-induced liver injury, p66Shc, a master regulator of mtROS, is upregulated in liver tissues in response to APAP. circ-CBFB acts as the sponge of miR-185-5p, triggering mitochondrial dynamics perturbation via p66Shc [95]. For the hydrogen peroxide (H_2_O_2_)-induced injury, circRNA-4099 augments H_2_O_2_-induced damage by inhibiting miR-706 and triggering Keap1/Nrf2 and p38MAPK in the L02 cells [96]. For anti-tuberculosis drug-induced liver injury, circMARS may participate in the compensation and regeneration of the liver through the circMARS–miR-6808-5p/miR-6874-3p/miR-3157-5p–KMT2C–EGFR functional axis [97].

For infection-induced liver injury, the mmu_circRNA_005186/miR-124-3p/Epha2 axis is associated with LPS-induced inflammation, and silencing mmu_circRNA_005186 attenuates this kind of inflammation [98]; circ_0003420 also mediates LPS-induced cellular injury and inflammation, mainly by targeting the neuronal PAS domain protein 4 (NPAS4) mRNA [99].

The current knowledge mentioned above is somewhat about the critical role of circRNAs in liver injury and liver regeneration. However, further studies that deepen the underlying mechanisms in these liver injuries are still urgently needed.

### 2.5. Liver Fibrosis/Cirrhosis

During chronic liver diseases, persistent inflammatory responses and parenchymal injury eventually lead to liver fibrosis and liver cirrhosis [100,101]. Chronic loss of hepatocytes occurs in chronic liver diseases of any etiology and is associated with the activation of HSCs and an abnormal liver microenvironment [6]. Thus, inhibiting HSC activation has been considered as one of the most potent approaches to alleviate the progression of liver cirrhosis.

Many circRNAs have been found to suppress the activation of HSCs. Telomerase-associated protein 1 (TEP1), a component of telomerase and target of hsa-miR-660-3p [102], plays a key role in the development of liver fibrosis and cirrhosis [103]. Overexpression of hsa_circ_0004018, a sponge of hsa-miR-660-3p, increases the expression of TEP1 and significantly suppresses the proliferation and activation of HSCs [104]. Consistently, hsa_circ_0007874 (cMTO1) could inhibit HSC activation through sponging miR-181b-5p and miR-17-5p [105,106]. Besides, hsa_circ_0070963 inhibits liver fibrosis via the regulation of miR-223-3p, which is related to HSC activation [107]. By targeting miR-18b-3p, circFBXW4 attenuates murine liver fibrogenesis with an anti-inflammation effect via inhibiting the activation and proliferation of HSCs, and inducing HSC apoptosis [108]. Moreover, mmu_circ_34116 can also inhibit the HSC activation via mmu_circ_34116/miR-22-3P/BMP7 signal axis [109,110]. Recently, circPSD3 was found to inhibit the activation and proliferation of HSCs by targeting the miR-92b-3p/Smad7 axis [111].

As mentioned above, these circRNAs keep HSCs in quiescent status. However, there are still some circRNAs that promote the activation of HSCs through different mechanisms. Follistatin-like 1 (Fstl1) contributes to TGF-β-induced HSC proliferation, the expression of α-SMA, and type I collagen, while TLR4 contributes to LPS-induced HSC activation [112]. circ-PWWP2A sponges miR-203 and miR-223, and subsequently increases the expression of Fstl1 and TLR4, respectively, eventually promoting the activation and proliferation of HSCs [112,113]. circRNA-0067835 acts as a sponge of miR-155 to promote FOXO3a expression, subsequently promoting fibrosis progression [56]. hsa_circ_0071410 decreases the expression of miR-9-5p, resulting in the progression of irradiation-induced HSC activation [114,115].

lncRNAs have also been shown to regulate the process of liver fibrosis by inhibiting miRNAs. For example, lncRNA-p21 can inhibit miR-181b and miR-17-5p, which in turn inhibit the activation of HSCs through two different signaling pathways [20]. Interestingly, both cMTO1 and lncRNA-p21 sponge miR-181b and miR-17-5p, which indicates the potential circRNA–miRNA–lncRNA network in the development of liver fibrosis.

Liver cirrhosis is a common ultimate consequence of chronic liver diseases. As HSCs are supposed to be a major source of collagen deposit during liver cirrhosis, the role of circRNAs in HSC activation attracts the most attention during chronic liver diseases. The hepatic cells are mainly composed of hepatocytes, LSECs, HSCs, cholangiocytes, and Kupffer cells, which precisely “cross-talk” to maintain normal hepatic functions and to aid cell survival [116]. Thus, the functions of circRNAs from other hepatic cells should also be investigated during chronic liver diseases and liver cirrhosis.

### 2.6. Autoimmune Liver Disease

Currently, the most common autoimmune liver diseases in clinical practice are primary biliary cholangitis (PBC), primary sclerosing cholangitis (PSC), and autoimmune hepatitis (AIH) [117]. They have a high heterogeneity of clinical presentation and result from the interactions of multiple genetic and environmental factors. circRNAs can contribute to the development of autoimmune diseases, mainly by serving as miRNA sponges to regulate DNA methylation, immune response and inflammatory response [118]. miR-522-3p might be involved in chronic inflammatory disorders, and miR-943 is a participant in the repair of DNA double-strand breaks [119]. Zheng et al. found that hsa_circ_402458, the sponge of hsa-miR-522-3p and hsa-miR-943, was significantly higher in PBC patients not treated with ursodeoxycholic acid (UDCA) than in those treated with it [119]. Liu et al. recently reported that the pathogenesis of AIH may involve the mmu_circ_0001520/mmu-miR-193b-3p/MAPK10 axis [120]. However, the role of circRNAs in PSC has not yet been reported. Overall, there are relatively few studies focusing on circRNAs and autoimmune liver disease. The etiology of autoimmune diseases is complex [121]. In different diseases, there is a significant overlap of susceptibility factors. Thus, the role of circRNAs in autoimmune liver disease may be complicated and would benefit from further study.

## 3. Conclusions and Prospects

**Figure 3:**
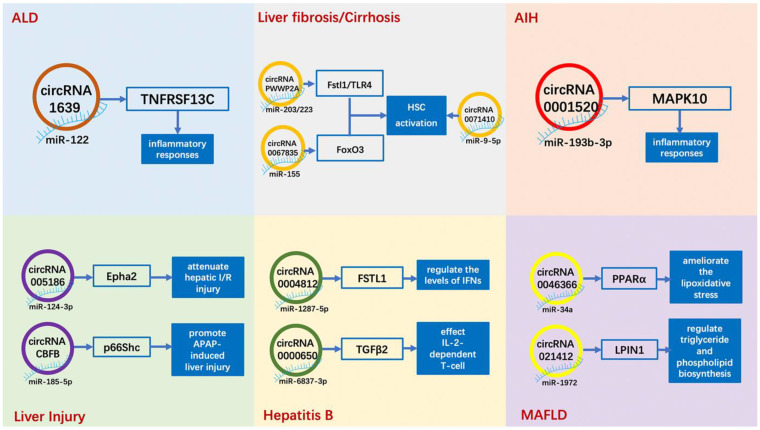
Examples of circRNAs in chronic liver diseases. ALD: alcoholic liver disease; MAFLD: metabolic-associated fatty liver disease; AIH: autoimmune hepatitis; HSC: hepatic stellate cell; I/R: ischemia/reperfusion; APAP: acetaminophen; IFN: interferon.

In this literature review, we discussed the epigenetic role of circRNAs in non-neoplastic chronic liver diseases (Table 1). Almost all current studies suggest that circRNAs act in chronic liver diseases through the ceRNA mechanism, i.e., through interactions with miRNAs, supporting a certain association between circRNAs and chronic liver diseases (Figure 3). Some miRNAs can interact with both circRNAs and lncRNAs in hepatic fibrosis, but in other chronic liver diseases, such intermediate miRNAs have not been reported.

However, the ceRNA network is more than just the interaction of circRNAs and miRNAs. Expressed 3′ untranslated regions (3′ UTRs), pseudogenes and lncRNAs may also act as ceRNAs to sequester miRNA activity by competing for miRNA binding sites. Investigating further connections among circRNA, miRNA and lncRNA in liver diseases could be a promising research direction in the future. Besides, circRNAs can be obtained from tissues, serum, and exosomes. The expression and function of circRNAs in the sites mentioned above may vary due to the different microenvironments. The clearance of circRNAs can be seen under a possible mechanism that is eliminating circRNAs from cells into the extracellular space by extracellular vesicles [24,25]. However, there are few studies on the role and function of circRNAs in serum and exosomes. Research on large samples from different sources is warranted to understand these circRNAs further.

Another important part of epigenetics is m^6^A modification. m^6^A modification plays a unique role in critical physiological hepatic functions and various liver diseases. It participates in glucose and lipid homeostasis involving the development of MAFLD and liver cancer pathogenesis and metastasis [122,123]. m^6^A is the most abundant internal modification known on DNA, mRNAs and lncRNAs [124,125]. Almost all studies have focused on the effects of m^6^A and mRNA, miRNA or lncRNA in MAFLD, liver fibrosis, PBC, and HCC [45,123,126,127,128,129]. m^6^A is widespread in circRNAs [130], regulating circRNA translation and facilitating circRNA degradation [131]. However, the relationship between m^6^A and circRNAs in chronic liver diseases remains unclear. Recently, Zhou et al. defined thousands of m^6^A circRNAs with cell-type-specific expression [130]. Although much of the same cellular machinery is shared between m^6^A-modified circRNAs and mRNAs, m^6^A circRNAs exhibit distinct patterns of m^6^A modifications compared with mRNAs. The discovery of m^6^A circRNAs raises many questions that will need to be addressed in future studies.

Liver diseases are a heavy burden on healthcare and account for significant costs worldwide. Clinicians and public health professionals may be more interested in how to use the characteristics of circRNAs and their interconnections with other molecules to diagnose and treat liver diseases. The articles published so far only provide new theories or ideas from the perspective of basic research, and there is still a long way to go before any clinical application. Therefore, it is worthwhile to reveal the full biological relevance of circRNAs and their potential therapeutics.

In conclusion, circRNAs play an increasingly crucial role in the epigenetic regulation of the onset and progression of chronic liver diseases. Further efforts are urgently needed in order to develop circRNAs as novel diagnostics and therapeutics.

## Figures and Tables

**Table 1:** Summary of circRNAs involved in chronic liver diseases.

Diseases	CircRNA or LncRNA	Possible Mechanisms	Expression Change	Refs.
ALD	circRNA_1639	circ_1639/miR-122/TNFRSF13C axis and NF-κB pathway	up-regulation	[49]
	mou_circ_1657	sponge miR-96-5p	up-regulation	[54]
MAFLD	circScd1	JAK2/STAT5 pathway	down-regulation	[66]
	circRNA_0049392	target miR-7037-5p and miR-6919-5p	up-regulation	[67]
	circRNA_0046366, circRNA_0046367	circRNA_0046366,7/miR-34a/PPAR α axis	down-regulation	[68,69]
	circRNA_021412	circRNA_021412/miR-1972/LPIN1 axis	down-regulation	[65]
	circRNA SCAR	shut down mPTP by binding to ATP5B	down-regulation	[62]
Viral hepatitis	hsa_circ_0004812	circ_0004812/miR-1287-5p/FSTL1 axis	up-regulation	[77]
	hsa_circ_0000650	hsa_circ_0000650/miR-6873-3p and TGF β 2 axis	down-regulation	[79]
	circPSD3	bind factor eIF4A3; nonsense-mediated decay (NMD) pathway	up-regulation	[83]
Liver injury and regeneration	circ432, circ2077, circ1366 and circ15	controlling the expression level of *MAPK14*, *FN1*, *TNFRSF21* and *GOT1*	up-regulation	[88]
	circRNA_017753	circRNA_017753/miR-218-5p, miR-7002-3p, miR-7008-3p/Jade1	up-regulation	[92]
	circRSF1	promoting inflammatory and fibrotic phenotypes of HSC by sponging miR-146a-5p	up-regulation	[93]
	circTUBD1	circTUBD1/miR-146a-5p/Toll-like receptor 4/NF-κB axis	up-regulation	[94]
	circ-CBFB	circ-CBFB/miR-185-5p/p66Shc axis	up-regulation	[95]
	circRNA-4099	circRNA-4099/miR-706/keap1/Nrf2 and p38MAPK axis	down-regulation	[96]
	circMARS	circMARS—miR-6808-5p/-6874-3p/-3157-5p—KMT2C—EGFR functional axis	up-regulation	[97]
	mmu_circRNA_005186	mmu_circRNA_005186/miR-124-3p/Epha2 axis	up-regulation	[98]
Liver fibrosis/Cirrhosis	hsa_circ_0004018	hsa_circ_0004018/hsa-miR-660-3p/TEP1 axis; inhibits liver fibrosis	down-regulation	[104]
	hsa_circ_0007874 (cMTO1)	inhibit HSC activation through sponging miR-181b-5p and miR-17-5p	down-regulation	[105,106]
	hsa_circ_0070963	inhibits liver fibrosis via regulation of miR-223-3p that related to HSC activation	down-regulation	[107]
	circFBXW4	circFBXW4/miR-18b-3p/FBXW7 axis; inhibit fibrosis	down-regulation	[108]
	mmu_circ_34116	mmu_circ_34116/miR-22-3P/BMP7 signal axis; inhibit HSC activation	down-regulation	[109,110]
	circPSD3	circPSD3/miR-92b-3p/Smad7	down-regulation	[111]
	circ-PWWP2A	circ-PWWP2A/miR-203 and miR-223/Fstl 1 and TLR4 axis; promote HSC activation	up-regulation	[112,113]
	circRNA-0067835	circRNA-0067835/miR-155/FoxO3 axis; promote HSC activation	up-regulation	[56]
	hsa_circ_0071410	sponge miR-9-5p; promote irradiation-induced HSC activation	up-regulation	[114,115]
Autoimmune liver disease	hsa_circ_402458	sponge hsa-miR-522-3p and hsa-miR-943	up-regulation	[119]
	mmu_circ_0001520	mmu_circ_0001520/mmu-miR-193b-3p/MAPK10	up-regulation	[120]
